# Supplementary material for: To eliminate trachoma: Azithromycin mass drug administration coverage and associated factors among adults in Goro district, Southeast Ethiopia
Source: PLoS Negl Trop Dis. 2022 Jun 27;16(6):e0010169. doi: 10.1371/journal.pntd.0010169 (PMC9236244; doi:10.1371/journal.pntd.0010169)
Supplement: S1 Fig — (DOCX) [file pntd.0010169.s001.docx]

Goro District (29 Kebeles)

Urban Kebeles (1Urban /3)

Rural kebeles (8 rural kebeles/26)

G/nagaye (Got=1, 2, 3)

B/akiya (Got=1, 2, 3)

B/gadula (Got=1, 2, 3)

Fankel (Got=1, 2, 3)

W/gobu (Got=1, 2, 3)

W/eltoke (Got=1, 2, 3)

W/Sayida (Got=1, 2, 3)

Keku (Got=1, 2, 3)

Maliyu town (Got=1, 2, 3, 4)

**Got1=24, Got 2=29**

**Got4=21,**

**=(74/HH)**

**Got2=21Got1=12**

**=(33HH)**

**Got1 =33 Got3=23,**

**=(56HH)**

**Gox3=47 Got1=35**

**= (82HH)**

**Got1=32**

**Got=45,**

**=(77HH)**

**Got3=44, Got 2=31**

**=(75HH)**

**Got1=22Got3=47**

**=(69HH)**

**Gox3=28.**

**Got2=23**

**=(51HH)**

**Got2=42 Got3=34**

**=(76HH)**

Go

519 HH from Rural Kebeles

74 HH from Urban Kebele

A total of 593 HH

**S1 Fig: Schematic presentation of sampling procedure to assess azithromycin mass drug administration uptake and associated factors in Goro district, Bale Zone, Southeast Ethiopia, 2021.**
