# Supplementary material for: To eliminate trachoma: Azithromycin mass drug administration coverage and associated factors among adults in Goro district, Southeast Ethiopia
Source: PLoS Negl Trop Dis. 2022 Jun 27;16(6):e0010169. doi: 10.1371/journal.pntd.0010169 (PMC9236244; doi:10.1371/journal.pntd.0010169)
Supplement: Attachment — Submitted filename: Responses to review comments.docx [file pntd.0010169.s002.docx]

**To eliminate trachoma: azithromycin mass drug administration coverage and associated factors among adults in Goro district, southeast Ethiopia**

Dear Academic Editor and Reviewers, Thank you for your devotion to your constructive comments and suggestion, which enrich our manuscript. The authors are so thrilled to receive a decision letter (Revised).

**Editor comment:** We would like to invite the resubmission of a significantly-revised version that takes into account the reviewers' comments.

**Authors’ response:** Dear Editor, Thank you! The authors responded to all the concerns raised by reviewers point by point, based on their understanding.

Reviewer's Responses to Questions

<b>Key Review Criteria Required for Acceptance?</b></br></br>

As you describe the new analyses required for acceptance, please consider the following:</br></br>
<b>Methods</b></br></br>
-Are the objectives of the study clearly articulated with a clear testable hypothesis stated?</br>

-Is the study design appropriate to address the stated objectives?</br>

-Is the population clearly described and appropriate for the hypothesis being tested?</br>

-Is the sample size sufficient to ensure adequate power to address the hypothesis being tested?</br>

-Were correct statistical analysis used to support conclusions?</br>

-Are there concerns about ethical or regulatory requirements being met?</br></br>
Reviewer #1: The methods presented here are straight forward and generally appropriate for the data. I do however have a few concerns and suggestions:

**Reviewer comment:** 1) If the analysis wasn’t weighted, then there is a bias in the results favoring responses from individuals living in households with few adults because the team randomly selected only one adult per household.  If the study team collected data on the number of adults in each household then they can weight the analysis appropriately (by using 1 / [number of adults in the HH] as the weight).  If this cannot be done, then it is important to be honest about this bias. If household size is associated with coverage then the findings are not representative.

**Authors’ response:** Thank you for your comment. In our study, there was no proportional allocation considered at the HH level. For each only one eligible participant was selected. As a result, weighted analysis is not considered necessary. Thank you again.

**Reviewer comment:** 2) Knowledge was deemed to be ‘good’ if the respondent scored points higher than the mean, but this is not a sign of good knowledge but rather a sign of knowing more than most people around you.  If the entire population has low knowledge, scoring above the mean is not a sign that one has good knowledge.  It would make more sense to me to set a threshold above which anyone scoring would be considered to have good knowledge (e.g. if you answer X or more of the questions correctly).  Alternatively, the team could leave the analysis as it has been done and replace good vs. bad knowledge with ‘better’ vs. ‘worse’ – this is more correct because it suggests that knowledge is being assessed relatively between two groups.

**Authors’ response:** Thank you for your comment. In this study, the cut point for the knowledge level was the mean of the scores. And, depending on the comments, the knowledge level was refined with better and worse knowledge throughout the manuscript. Thank you again.

**Reviewer comment:** 3) Did the analysis account for clustering at the Kebele level?  Since a design effect was included in the sample size calculations, it suggests that the analysis intends to account for correlation within clusters. It is important to specify if and how this was done, as many previous studies have shown high correlation within clusters when it comes to mass treatment coverage.

**Authors’ response:** Thank you for your comment. Cluster sampling at Kebele level was not employed in this study. First, kebeles were divided into rural and urban strata using stratification. The researchers employed a multi-stage stratified sampling technique, with stage one consisting of selecting Kebeles from the district, stage two consisting of selecting households from the Kebeles, and stage three consisting of selecting adult aged ≥ 15 years old from the homes. As a result, a design effect of 1.5 was considered for increasing the sample size to address sampling error. Thank you again.

Reviewer #2:

**Reviewer comment:** Source and study population, inclusion criteria as well as exclusion criteria were not stated.

**Authors’ response:** Thank you for your comment. The authors encompassed the source, study population, inclusion and exclusion criteria based on the comment. *(Highlighted blue on page #7, line #142-146).* Thank you again.

Reviewer #3:

**Reviewer comment:** The authors conducted a community-based cross-sectional study using a structured interviewer-administered questionnaire.

The objectives of the study were clearly articulated, with an appropriate study design.
They clearly described how the sample size was estimated, and the statistical analysis used to support conclusions.

There are no concerns about ethical or regulatory requirements in the study.

**Authors’ response:** Thank you for your comment. We appreciate your comments kindly.

--------------------

<b>Results</b></br></br>
-Does the analysis presented match the analysis plan?</br>

-Are the results clearly and completely presented?</br>

-Are the figures (Tables, Images) of sufficient quality for clarity?</br></br>

Reviewer #1: I would like to suggest the following revisions:

**Reviewer comment:** 1) It is important to be consistent with the use of significant figures (ie, how many numbers are reported after the decimal) in all the numbers (CIs, %s, means, etc.) that are reported in the tables and text.

**Authors’ response:** Thank you for your comment! We have corrected reporting of figures regarding Odds ratio, % and mean…by two digits after decimal point throughout the manuscript document. Thank you again.

**Reviewer comment:** 2) Table 1 – formatting is off, some of the variable names in Column 1 appear to be centered in the cell while others appear to be at the top of the row, which makes for difficult reading.

**Authors’ response:** Thank you for your comment. Table 1 formatting was modified making column 1 at the top of the row. *(Corrected on page #12-13).* Thank you again.

**Reviewer comment:** 3) Figure 3 – Please add the counts to this pie chart and correct the typos in the boxes.

**Authors’ response:** Thank you for your comment. Counts were added to all pie charts throughout the manuscript and corrected according to the comment. *(Corrected on page #13& 16).* Thank you very much.

**Reviewer comment:** 4) I don’t see the crude results for ‘conducting campaigns at the quietest time’; please include this information in Table 1 or one of the later tables.

**Authors’ response:** Thank you for your comment. We have corrected this issue as per the given comment. *(Highlighted blue on page# 19, lines #326-328).* Thank you once more.

Reviewer #2:

**Reviewer comment:** The results should be stated in line to the objectives

**Authors’ response:** Thank you for your comment. The authors refined the result section based on the comment. Thank you once more.

Reviewer #3:

**Reviewer comment:** The analysis presented matched the analysis plan.

The results were clearly and completely presented.

The figures (Tables, Images) are of sufficient quality for clarity.

**Authors’ response:** Thank you for your comment. We appreciate your comments kindly.

--------------------

<b>Conclusions</b></br></br>
-Are the conclusions supported by the data presented?</br>

-Are the limitations of analysis clearly described?</br>

-Do the authors discuss how these data can be helpful to advance our understanding of the topic under study? </br>

-Is public health relevance addressed? </br></br>

Reviewer #1: The authors do not adequately describe some key limitations of the study and there is room for greater discussion on the implications of these findings. I would like to suggest that they address the following in the discussion:

**Reviewer comment:** 1) How complete is the Community Health Information System, in terms of its inclusion of all households? Is this list kept up-to-date locally? Is there a risk that some new households, ethnic minorities, or other marginal groups might be missing?

**Authors’ response:** Thank you for your comment. The district (Goro district one of model district in employing CHIS) is implementing the Community Health Information System (CHIS), which includes health post Family Folder that is updated and complete on regular basis. In this context, CHIS Family Folder is a tool or package designed to be used for data collection and documentation to meet the necessary information, to promote family-centered health care at the community level. Through a procedure known as 'kebele profiling,' all of the households in the kebeles were included in CHIS; as a result, there is no risk of missing ethnic minorities or marginalized groups in the kebele. Subsequently, the kebele profile is updated every year based on updated data recorded on the Family Folders.

Besides, data on Family Folder is updated routinely; when service is provided at health post, during household visit, and during outreach service delivery by Health Extension Workers. Meanwhile, new households also included. Thank you very much again.

**Reviewer comment:** 2) One potential bias of this study is that the respondents were limited to people who were home at the time of the survey team’s visit. How might this impact your results? I suggest you comment on this in the discussion.

**Authors’ response:** Thank you for comment. In our study, once household was selected eligible participant were randomly chosen, in case selected participant was not found at home during data collection revisit was done on next day, after appointing him/her. Thank you again.

**Reviewer comment:** 3) Line 358 – I don’t understand this statement. The current study did not indicate anything about reinfection. If the authors are referring to reference 26, then then need to be more clear in the opening of the sentence that they are referring to another study.

**Authors’ response:** Thank you for your comment. Definite! We didn't look at reinfection in our research. The statement is from reference #26; we rewrote the wording in light of the feedback. *(Highlighted blue on page #23, line 378-380).* Thank you again.

**Reviewer comment:** 4) The fact that willingness to comply with treatment in future rounds was so significant is potentially worrisome to trachoma programs. It suggests that there may be strong systematic compliance (and conversely systematic non-compliance) whereby the same group in the population is routinely participating while others are not. It would be good to understand what would be required to get the non-participants to participate in the future. Perhaps adding the question, “what would it take for you to participate in future rounds of AMT?” would be beneficial to add to a future study.

**Authors’ response:** Exactly! The question, “what would it take for you to participate in future rounds of AMT?” needs to be taken in to account in the future studies. Accordingly, we distinguished this in our recommendation for future studies based on the comment. *(Highlighted blue on page #27, line 457-459).*Thank you again.

**Reviewer comment:** 5) The discussion would be strengthened if, rather than just point out all the places where this study’s findings agree with past studies, the authors also added in implications for the trachoma program and recommendations for how to improve implementation of AMT and achieve higher coverage.

**Authors’ response:** Thank you for your comment. We have refined the discussion section based on the comment. Thank you again.

Reviewer #2:

**Reviewer comment:** How the current coverage of the AMT 75.8% inline to WHO recommendation (80%)?

**Authors’ response:** Thank you for your comment**.** The AMT coverage finding in this study (75.80%) is indeed lower than that of WHO recommendation (80%). The comment well considered and corrected in the manuscript. Thank you again**.**

Reviewer #3:

**Reviewer comment:** The conclusions were supported by the data presented.

The authors highlighted the need to strengthen social mobilization about the Azithromycin Mass Treatment campaign and due consideration of additional campaigns at the non-harvesting nor the planting time for other trachoma endemic areas.

**Authors’ response:** Thank you for your comment. We appreciate your comments kindly.

--------------------
<b>Editorial and Data Presentation Modifications?</b></br><br/>

Use this section for editorial suggestions as well as relatively minor modifications of existing data that would enhance clarity. If the only modifications needed are minor and/or editorial, you may wish to recommend “Minor Revision” or “Accept”.

Reviewer #1:

**Reviewer comment:** This study needs a very close proof read by a native English speaker. There are too many grammatical errors throughout the paper for me to comment on here. Many have to do with the use of prepositions and noun agreement.

**Authors’ response:** Thank you for your comment. The grammatical errors critically review and refined throughout the manuscript. Thank you again.

Reviewer #2:

Abstract

**Reviewer comment:** The background lacks aim of the study

**Authors’ response:** Thank you for your comment. The main aim of this study incorporated in the background section of the abstract. *(Highlighted blue on page #2, line 29-30).* Thank you again.

Methods:
**Reviewer comment:** Line 37: Principal finding should be expunged.

**Authors’ response:** Thank you for your comment. The result section stated as Principal Findings is in order to stick to the journal guideline. Thank you again.

Results
**Reviewer comment:** Which one is appropriate for your study? Prevalence or magnitude?

**Authors’ response:** Thank you for your comment. In our context, we can use Prevalence and Magnitude interchangeably. However, prominently, magnitude is preferable to show the coverage level of AMT uptake. Thank you again.

Conclusion
**Reviewer comment:** Line 47: Significance should also be expunged.

**Authors’ response:** Thank you for your comment. The conclusion as well stated as Significance is in order to follow the journal guideline. Thank you again.

**Reviewer comment:** How the current finding 75% in line to WHO recommendation (80%)

**Authors’ response:** Thank you for your comment. The AMT coverage finding in this study (75%) is indeed lower than that of WHO recommendation (80%). The comment well considered and corrected in the manuscript. Thank you once more.

**Reviewer comment:** Keywords: Add coverage

**Authors’ response:** Thank you for your comment. We have included ‘coverage’ in keywords. *(Highlighted blue on page 3, line 55).*

Main body

Introduction
**Reviewer comment:** intervention measures to mitigate the problems should be stated after the rationale of the study were indicated

**Authors’ response:** Thank you for your comment. We have refined this section based on the comment. Thank you again.

Methods

**Reviewer comment:** Line 122: Rearrange it

**Authors’ response:** Thank you for your comment. We have rearranged this sub-heading in the manuscript, particularly enlisting the ‘Population’ in the distinct sub-heading. *(Highlighted red on page #7, line #127).* Thank you again.

**Reviewer comment:** Source and study population, inclusion criteria as well exclusion criteria were not specified.

**Authors’ response:** Thank you for your comment. The authors encompassed the source, study population, inclusion and exclusion criteria based on the comment. *(Highlighted blue on page #7, line #142-146).* Thank you again.

**Reviewer comment:** Outcome assessment should be referenced.

**Authors’ response:** Thank you for your comment. The authors cited the references for the outcome measurement. *(On page #9, line #187).* Thank you again.

**Reviewer comment:** Why did the authors use mean for knowledge question assessment?

**Authors’ response:** Thank you for your comment. We asked respondents nine knowledge assessment questions. And, we found the knowledge score normally distributed, and the mean score was considered as a cut-off point to measure the knowledge level. Thank you again.

**Reviewer comment:** Have you checked intervention of variables and identify confounding factors?

**Authors’ response:** Thank you for your comment. We identified and controlled for confounding factors through a multivariable logistic regression model. Thank you again.

**Reviewer comment:** State why did the authors use multivariable LR analysis?

**Authors’ response:** Thank you for your comment. Multivariable regression models are used to establish the relationship between a dependent variable (i.e. an outcome of interest) and more than 1 independent variable, and to control for confounding variables effect i.e. often with the effect of other predictors statistically eliminated. Thank you again.

**Reviewer comment:** Restate line 215-216.

**Authors’ response:** Thank you for your comment. We have rephrased this statement based on the comment. *(On page #11, lines #229-230).* Thank you again.

**Reviewer comment:** Line 217- 218 should be written after line 217.

**Authors’ response:** Thank you for your comment. We have restated these statements based on the comment. *(Highlighted green on page #11, lines #228-229).* Thank you again.

**Reviewer comment:** State how did you assess normality of the data distribution?

**Authors’ response:** Thank you for your comment. We have conducted a normality test in the statistical software “SPSS” (analyze → descriptive statistics → explore → plots → normality plots with tests). Tests of normality (Kolmogorov–Smirnov test and the Shapiro–Wilk test), Q-Q plot, and Boxplot were evaluated and then the normality of data was interpreted. Thank you again.

Ethical consideration

**Reviewer comment:** State tenet of Helsinki Declaration

**Authors’ response:** Thank you for your comment. We have incorporated the Helsinki Declaration protocol in our ethical declaration. *(Highlighted blue on page #11, line #234).* Thank you again.

Results
**Reviewer comment:** Line 227: Restate as ' Sociodemographic and economic characteristics of ...

**Authors’ response:** Thank you for your comment. We have refined this heading based on the comment. *(Highlighted blue on page #12, line #242).* Thank you again.

**Reviewer comment:** Substitute majority by most throughout the results part

**Authors’ response:** Thank you for your comment. We have substituted ‘majority’ by ‘most’ throughout the result section. Thank you again.

**Reviewer comment:** Line 34-316: should be expunged as they are not the objective of the study.

**Authors’ response:** Thank you for your comment. We have removed table #3 and its contents as this is not the primary objective of the study. *(Highlighted red on pages #19-20).* Thank you again.

**Reviewer comment:** Line 262: should be merged with table 2.

**Authors’ response:** Thank you for your comment. We refined this based on the comment. Thank you again.

Discussion
**Reviewer comment:** The discussion is clumsy.

**Authors’ response:** Thank you for your comment. We have refined the discussion section based on the comment. Thank you again.

**Reviewer comment:** Line 345: Did the CI overlap each other to use the word "comparable"?

**Authors’ response:** Thank you for your comment. Definitely! Our study CI i.e. 95% CI: (72%-79%) encompasses those studies results we stated as comparable. Thank you again.

**Reviewer comment:** Limitation of the study was not indicated

**Authors’ response:** Thank you for your comment. The authors well declared possible limitation of the study. *(Highlighted blue on page #26, lines #444-445).* Thank you again.

Conclusion

**Reviewer comment:** As commented in the abstract section

**Authors’ response:** Thank you for your comment. We have addressed this comment well in the manuscript. Thank you again.

Reviewer #3:

**Reviewer comment:** Minor comment:

**Authors’ response:** Thank you very much for your comment.

**Reviewer comment:** Correct the figure number in the legends for figures 2-4.

**Authors’ response:** Thank you for your comment. We have corrected based on the comment. Thank you again.

**Reviewer comment:** Rephrase the last sentence, starting from line 425, to clarify the confusing language.

**Authors’ response:** Thank you for your comment. We have rephrased this statement based on the comment. *(Highlighted blue on page #26-27, lines #453-457).* Thank you again.

--------------------
<b>Summary and General Comments</b></br></br>

Use this section to provide overall comments, discuss strengths/weaknesses of the study, novelty, significance, general execution and scholarship. You may also include additional comments for the author, including concerns about dual publication, research ethics, or publication ethics. If requesting major revision, please articulate the new experiments that are needed.

Reviewer #1:

**Reviewer comment:** This article presents the results of a coverage survey for azithromycin mass treatment in Ethiopia.  I would like to start by applauding the study's authors for undertaking this work. Coverage surveys are an important monitoring tool to help NTD programs understand how the implementation of the program and may be used to identify important gaps in treatment and areas for improvement. Unfortunately, coverage surveys are not routinely conducted by most trachoma programs and thus this study is a good one to add to the evidence base.

The team appears to have done a diligent job making sure the questionnaire was well-understood, appropriate, and completed for each respondent. I appreciate the inclusion of a dedicated pre-test and refinement period.

It is very interesting, and perhaps reassuring, that ‘Azithromycin not offered’ or ‘Nobody came’ were not among the reasons mentioned for not taking the drug.  This suggests that the majority of the challenges with coverage in this district are related to the participants (both their physical presence and psychological reasoning) and not the distributors or supply chain.

**Authors’ response:** Thank you for your comment. We kindly appreciate your comments.

Reviewer #2:

**Reviewer comment:** The aim of the study should be specified in the abstract.

**Authors’ response:** Thank you for your comment. We have refined this section based on the comment. *(Highlighted blue on page #2, line 29-30).* Thank you again.

**Reviewer comment:** Source and study population, inclusion criteria as well exclusion criteria were should be specified.

**Authors’ response:** Thank you for your comment. The authors encompassed the source, study population, inclusion and exclusion criteria based on the comment. *(Highlighted blue on page #7, line 142-146).* Thank you again.

**Reviewer comment:** Outcome assessment should be cited.

**Authors’ response:** Thank you for your comment. The authors cited the references for the outcome measurement. *(Corrected on page #9, line #187).* Thank you again.

**Reviewer comment:** Why did the authors use mean for knowledge question assessment?

**Authors’ response:** Thank you for your comment. We asked respondents nine knowledge assessment questions. And, we found the knowledge score normally distributed, and the mean score was considered as a cut-off point to measure the knowledge level. Thank you again.

Reviewer #3:

**Reviewer comment:** The study was well designed, analyzed and interpreted.

**Authors’ response:** Thank you for your comment. We appreciate your comment kindly. Thank you again.

-------------------
